# P53 is Subjected to Lipoteichoic Acid-Induced Phosphorylation in the Lungs

**DOI:** 10.1055/s-0040-1714695

**Published:** 2020-08-20

**Authors:** Khadeja-Tul Kubra, Mohammad A. Uddin, Mohammad Shohel Akhter, Nektarios Barabutis

**Affiliations:** 1 School of Basic Pharmaceutical and Toxicological Sciences, College of Pharmacy, University of Louisiana Monroe, Monroe, Louisiana, United States

P53 is a transcription factor protecting the cells against malignancies via modulation of multifarious regulatory signaling cascades. Those activities may result to either cellular repair, or to the elimination of the irreversible damaged tissue components. Recent evidence suggest that this endothelium defender (P53) exerts strong anti-inflammatory activities in the lungs. 1 P53 protects the endothelium cells against the lipopolysaccharide (LPS)-induced endothelial hyperpermeability by reducing the generation of the reactive oxygen species, 2 by suppressing the inflammatory RhoA/MLC2 pathway, 3 and by inducing the repairing activities of the unfolded protein response in the lungs. 4 5

Lung endothelial barrier dysfunction is both a cause and a consequence of severe lung inflammatory disease, including the lethal acute respiratory distress syndrome (ARDS). 1 Indeed, P53 expression levels are crucial for the integrity of the lung microvasculature, since P53 reduction due to LPS-induced P53 phosphorylation or small interfering ribonucleic acid has been previously shown to be related to the collapse of the lung barrier function. 6 Lipoteichoic acid (LTA) contributes in ARDS. 7

**Fig. 1:**
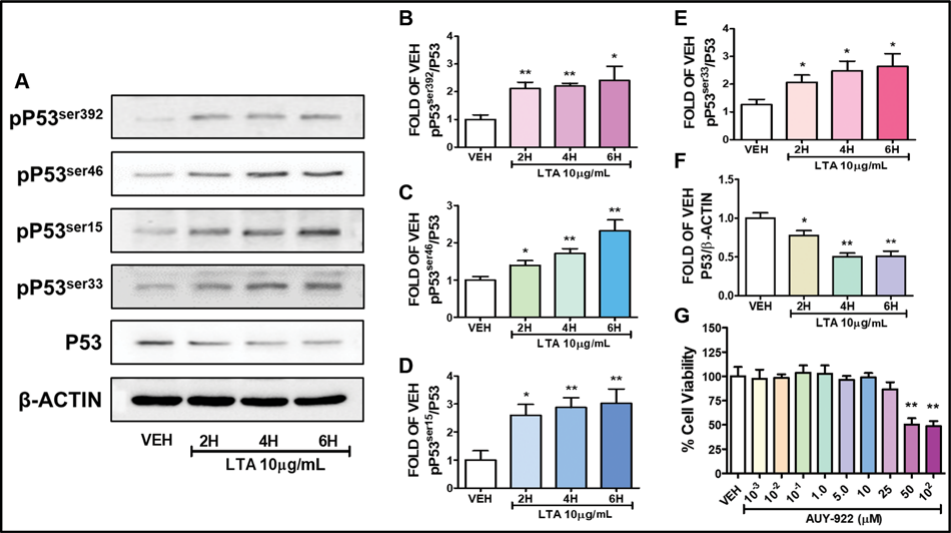
( **A** – **F** ) Western blot analysis of phosphorylated P53 (pP53 ^ser392^ , pP53 ^ser46^ , pP53 ^ser15^ , pP53 ^ser33^ ) and total P53 expression after treatment of bovine pulmonary artery endothelial cell (BPAEC) with either lipoteichoic acid (LTA) (10 µg/mL) or vehicle (VEH) (phosphate-buffered saline [PBS]) for 2, 4, and 6 hours. The blots shown are representative of three independent experiments. The signal intensity of the protein bands was analyzed by densitometry. Protein levels of phosphorylated P53 and P53 were normalized to P53 and β-actin, respectively. * *p* < 0.05, ** *p* < 0.01 vs. VEH. Means ± standard error of mean (SEM). ( **G** ) Effects of the Hsp90 inhibitor AUY-922 in the viability of HuLEC-5a. Cells were treated with either VEH (0.1% dimethyl sulfoxide [DMSO]) or AUY-922 (10 ^−3^ , 10 ^−2^ , 10 ^−1^ , 1.0, 5.0, 10.0, 25.0, 50.0, 100 µM) for 24 hours. Cell viability was evaluated by employing the 3-(4,5-dimethylthiazol-2-yl)-2,5-diphenyltetrazolium bromide (MTT) assay. ** *p* < 0.01 vs. VEH, *n* = 3. Means ± SEM.

Fig. 1A demonstrates by Western blotting in bovine pulmonary arterial endothelial cells purchased from Genlantis (PB30205) (San Diego, California, United States) that LTA induces the phosphorylation of P53 and suppresses its expression levels. The LTA from *Staphylococcus aureus* (L2515) was purchased from Sigma-Aldrich (St. Louis, Missouri, United States). The densitometric analysis performed with Image J software indicated that this toxin, which is a major constituent of the cell wall of Gram-positive bacteria, increases the expression of pP53 ^ser392^ ( Fig. 1B ), pP53 ^ser46^ ( Fig. 1C ), pP53 ^ser15^ ( Fig. 1D ), and pP53 ^ser33^ ( Fig. 1E ), and reduces P53 ( Fig. 1F ). Interestingly, Hsp90 inhibitors are anticancer agents, which have been shown to counteract the LPS-induced P53 degradation, and deliver protective effects in the inflamed lungs. 3 Although those compounds were initially developed to stochastically eliminate cancers, it now appears ( Fig. 1G ) that they do not affect the viability of human lung microvascular cells (HuLEC-5a) (CRL-3244), which were obtained from the American Type Culture Collection (Manassas, Virginia, United States). Details regarding cell cultures and Western blotting have been previously reported. 2 4

Treatment of HuLEC-5a cells with moderate concentrations of AUY-922 (101756–820) ( Fig. 1G ) from VWR (Radnor, Pennsylvania, United States) did not affect the viability of those cells, as measured with the 3-(4,5-dimethylthiazol-2-yl)-2,5-diphenyltetrazolium bromide (MTT) assay. Briefly, the cells were seeded in 96-well culture plates (10,000 cells/well) in complete growth media and were treated with AUY-922 (0–100 µM). After 24 hours, the media was replaced with fresh media containing 0.5 mg/mL MTT. After 3.5 hours of incubation, dimethyl sulfoxide (100 μL/well) was added to dissolve the MTT crystals, and 15 minutes later the absorbance was measured at 570 nm in a Synergy H1 Hybrid Multi-Mode Reader from Biotek (Winooski, Vermont, United States). In all cases, GraphPad Prism (version 5.01) was used to analyze the data, and the values are expressed as the mean ± standard error of mean. Values of *p* less than 0.05 were considered as an indication of statistical significance, and the number of experimental repeats is indicated by the letter *n* .

In conclusion, the present letter aims to substantiate our hypothesis that P53 is a target of the “inflammatory storm”-induced ARDS. Thus, pharmacological induction of P53 due to treatments with Hsp90 inhibitors, or growth hormone releasing hormone antagonists 8 may deliver a promising approach against the severe lung inflammatory disease. 9
